# Supporting the gastrointestinal microenvironment during high-dose chemotherapy and stem cell transplantation by inhibiting IL-1 signaling with anakinra

**DOI:** 10.1038/s41598-022-10700-3

**Published:** 2022-05-11

**Authors:** H. R. Wardill, C. E. M. de Mooij, A. R. Da Silva Ferreira, H. Havinga, H. J. M. Harmsen, W. J. F. M. van der Velden, L. F. J. van Groningen, W. J. E. Tissing, N. M. A. Blijlevens

**Affiliations:** 1 School of Biomedicine, The University of Adelaide, Adelaide, SA Australia; 2 The Supportive Oncology Research Group, Precision Medicine Theme (Cancer), The South Australian Health and Medical Research Institute, Adelaide, SA Australia; 3 Department of Pediatrics, The University of Groningen, University Medical Center Groningen, Groningen, The Netherlands; 4 Department of Hematology, Radboud University Medical Center, Nijmegen, The Netherlands; 5 Department of Medical Microbiology, The University of Groningen, University Medical Center Groningen, Groningen, The Netherlands; 6 Princes Maxima Center for Pediatric Oncology, Utrecht, The Netherlands

**Keywords:** Myeloma, Experimental models of disease, Quality of life, Translational research, Drug development, Phase II trials, Infection, Acute inflammation, Sepsis, Mucosal immunology, Interleukins, Pathogens, Diarrhoea

## Abstract

High-dose chemotherapy causes intestinal inflammation and subsequent breakdown of the mucosal barrier, permitting translocation of enteric pathogens, clinically manifesting as fever. Antibiotics are mainstay for controlling these complications, however, they are increasingly recognized for their detrimental effects, including antimicrobial resistance and dysbiosis. Here, we show that mucosal barrier injury induced by the mucotoxic chemotherapeutic agent, high-dose melphalan (HDM), is characterized by hyper-active IL-1b/CXCL1/neutrophil signaling. Inhibition of this pathway with IL-1RA, anakinra, minimized the duration and intensity of mucosal barrier injury and accompanying clinical symptoms, including diarrhea, weight loss and fever in rats. 16S analysis of fecal microbiome demonstrated a more stable composition in rats receiving anakinra, with reduced pathogen expansion. In parallel, we report through Phase IIA investigation that anakinra is safe in stem cell transplant patients with multiple myeloma after HDM. Ramping-up anakinra (100–300 mg administered intravenously for 15 days) did not cause any adverse events or dose limiting toxicities, nor did it change time to neutrophil recovery. Our results reinforce that strengthening the mucosal barrier may be an effective supportive care strategy to mitigate local and systemic clinical consequences of HDM. We are now conducting a Phase IIB multicenter, placebo-controlled, double-blinded trial to assess clinical efficacy of anakinra (AFFECT-2).

**Trial registration**: ClinicalTrials.gov identifier: NCT03233776.

## Introduction

Treatment of hematological malignancies with intensive cytotoxic therapy and hematopoietic stem cell transplantation (HSCT) is frequently complicated by the occurrence of mucosal barrier injury (MBI); an adverse event in which chemotherapy causes cytotoxic and inflammatory injury to the gastrointestinal mucosa. Earlier studies have shown MBI to be a major determinant of important inflammatory complications in HSCT recipients, most notably fever, bloodstream infections (BSI), and graft-versus-host disease (GvHD)^1–4^. These complications lead to significant morbidity and non-relapse mortality (NRM) and are related to substantial use of hospital resources and increased healthcare costs^4–7^. Moreover, the extensive use of prophylactic and empirical antibiotics in the prevention and treatment of BSI lead to disturbance of the microbiota (dysbiosis), which in turn is associated with the development of MBI, pulmonary complications, GvHD, and increased mortality in HSCT recipients^8–14^. Considering the globally expanding rates of antibiotic resistance, the current focus on antibiotic stewardship therefore addresses only a part of this problem with alternative methods of infection control desperately required^15, 16^.

First-line treatment of multiple myeloma with high dose melphalan (HDM) and autologous HSCT^17, 18^, is an exemplary clinical scenario in which MBI, fever and BSI frequently occur^19^. It can be used as a basic and comprehensible clinical model of MBI, in part due to the extensive experience with this regimen^1, 19, 20^. In the autologous setting of HDM, potential interventions can be tested, which may then be translated to more complex settings including allogeneic HSCT.

To explore methods of preserving the mucosal barrier, we recently developed a translational preclinical model of HDM-induced MBI^21^. This model recapitulates the complex etiology of MBI, where melphalan injures the gut microbiota and impairs the microbial metabolome, causing expansion and subsequent translocation of enteric pathogens across a damaged mucosal barrier. The goal of the current study is to identify mucosa-directed interventions to minimize MBI, as there are currently no approved strategies to prevent or control MBI^22^. While MBI is driven by extensive inflammation, evidence suggests that activation of the interleukin (IL) pathway is critical in initiating exacerbatory feedback loops which ultimately degrade the restrictive properties of the mucosal barrier. Anakinra (Kineret) is a recombinant IL-1 receptor antagonist (IL-1RA), which is used in a wide range of rheumatoid and autoimmune diseases^23, 24^, and has demonstrated efficacy in controlling MBI caused by other chemotherapeutic agents^25–27^. Although generally safe and well-tolerated, in rheumatology anakinra is associated with the occurrence of neutropenia, and an increased risk of infections during prolonged use^28, 29^. It also remains unclear if anakinra is effective in controlling MBI caused by melphalan, limiting its application to hematology where MBI is the most significant adverse effect. As such, the current study aimed to (1) determine the preclinical efficacy of anakinra to prevent HDM-induced MBI, related inflammatory complications and microbial disruption, and (2) determine the safety and maximum tolerated dose of anakinra in patients with multiple myeloma treated with HDM and autologous HSCT. We hypothesize that by targeting inflammatory mechanisms documented to exacerbate the intensity and duration of mucosal injury, we can minimize MBI, and may therefore have an impact on fever and infections ^30^. This would reduce the need for antibiotics, prevent further distortion of the microbiota, and consequently decrease the incidence of related inflammatory complications such as GvHD, and thus may decrease NRM.

## Results

### Preclinical study

In accordance with the ARRIVE guidelines, key baseline variables are shown in Table S1. All animals included in the study were retained for all downstream analyses.

#### Melphalan-induced MBI is driven by a hyper-reactive IL-1β/CXCL1/neutrophil axis

**Figure 1:**
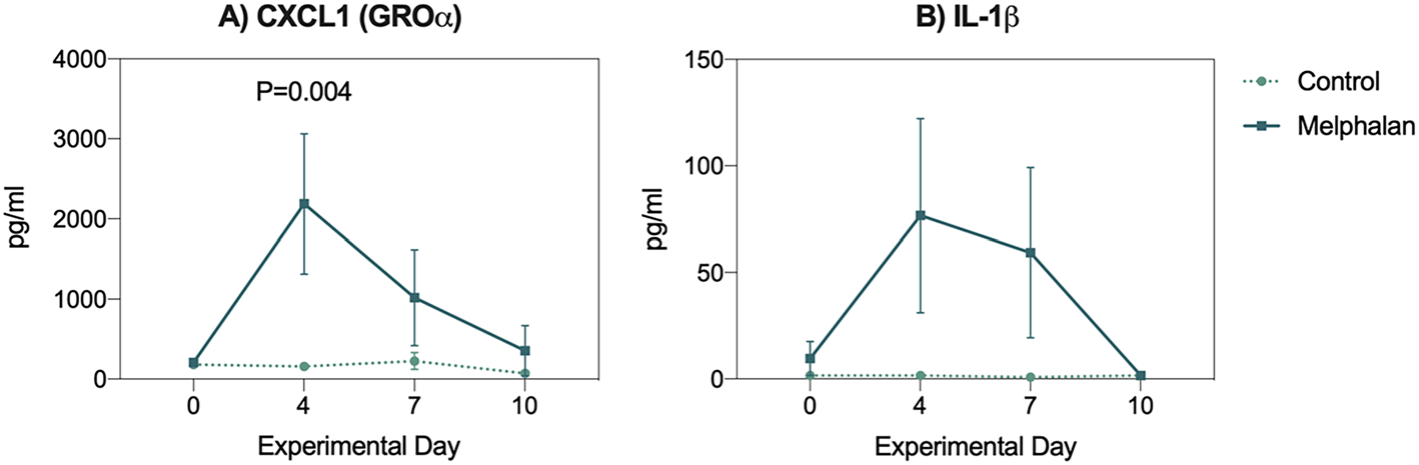
Melphalan causes hyperactivation of the CXCL1 (GROα) / IL-1β axis in rats. (**A**) CXCL1 and (**B**) Il-1β were assessed in repeated blood samples collected after melphalan treatment. Data presented as mean ± SEM. N = 8/group, all analyzed longitudinally using a mixed-effects model with Geisser-Greenhouse correction for multiple comparisons.

5 mg/kg melphalan caused a significant increase in the expression of C-X-C Motif Chemokine Ligand 1 (CXCL1, KC GRO in rats) 4 days post-treatment (159.1 ± 12.0 pg/ml vs 2186.8 ± 876.6 pg/ml, *P* = 0.004, Fig. 1A). In line with the regulatory interplay between CXCL1 and interleukin (IL)-1β, plasma IL-1β was increased at day 4 (Fig. 1B). This reinforced our selection of anakinra as suitable intervention to inhibit exacerbaory inibition and strengthen the mucosal barrier.

#### Anakinra mitigates MBI and clinical symptomology of melphalan-induced gut toxicity without affecting myeloablation

##### Clinical assessment

**Figure 2:**
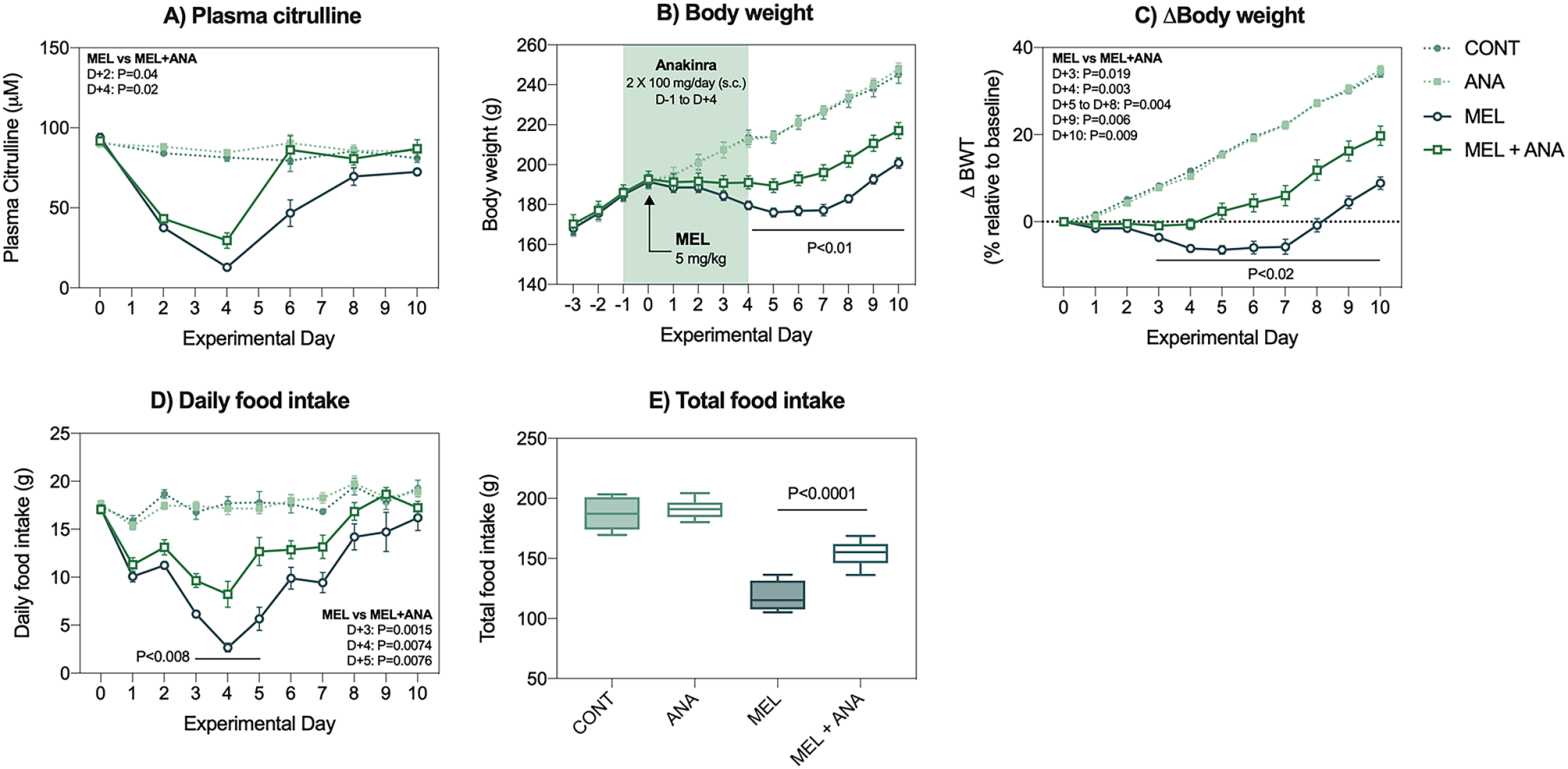
Anakinra mitigates melphalan-induced mucosal barrier injury and clinical manifestations of gut toxicity. (**A**) Mucosal barrier injury defined by plasma citrulline showed a significant decrease in MEL + ANA at D + 2 and D + 4 compared to melphalan only. (**B**–**C**) Anakinra prevented melphalan-induced weight loss and food intake (**D**–**E**). Data presented as mean ± SEM. N = 8/group, all analyzed longitudinally using a mixed-effects model with Geisser-Greenhouse correction, with the exception of panel (**E**) which used a one-way ANOVA with Tukey’s post-hoc.

To determine the impact of anakinra on melphalan-induced MBI, plasma citrulline—a biomarker of mucosal injury-was longitudiunally analysed. Intravenous melphalan caused a significant decrease in plasma citrulline (*P* < 0.0001 D2 and D4 compared to control and anakinra control, Fig. 1A) that returned to baseline levels within 10 days. While anakinra was unable to completely prevent melphalan-induced MBI, plasma citrulline during peak MBI was significantly improved in rats treated with MEL + ANA compared to MEL alone (D2: *P* = 0.041; D4: *P* = 0.022, Fig. 2A). To assess both the severity and duration of MBI, area under the curve (AUC) was calculated for all citrulline data. MEL + ANA plasma citrulline AUC was significantly lower compared to MEL only (416.7 ± 30.36 vs 258.1 ± 41.1, *P* = 0.005, data now shown).

Anakinra treatment significantly protected against melphalan-induced weight loss, with MEL + ANA animals losing a maximum of 0.91 ± 0.67% body weight on day 3. Body weight and change relative to baseline was significantly higher in MEL + ANA animals compared to MEL alone at day 4–10 (*P* < 0.04; Fig. 2B/C). Food intake was also improved in MEL + ANA animals (*P* < 0.0001 Fig. 2D/E).

##### Blood analysis and body temperature

**Figure 3:**
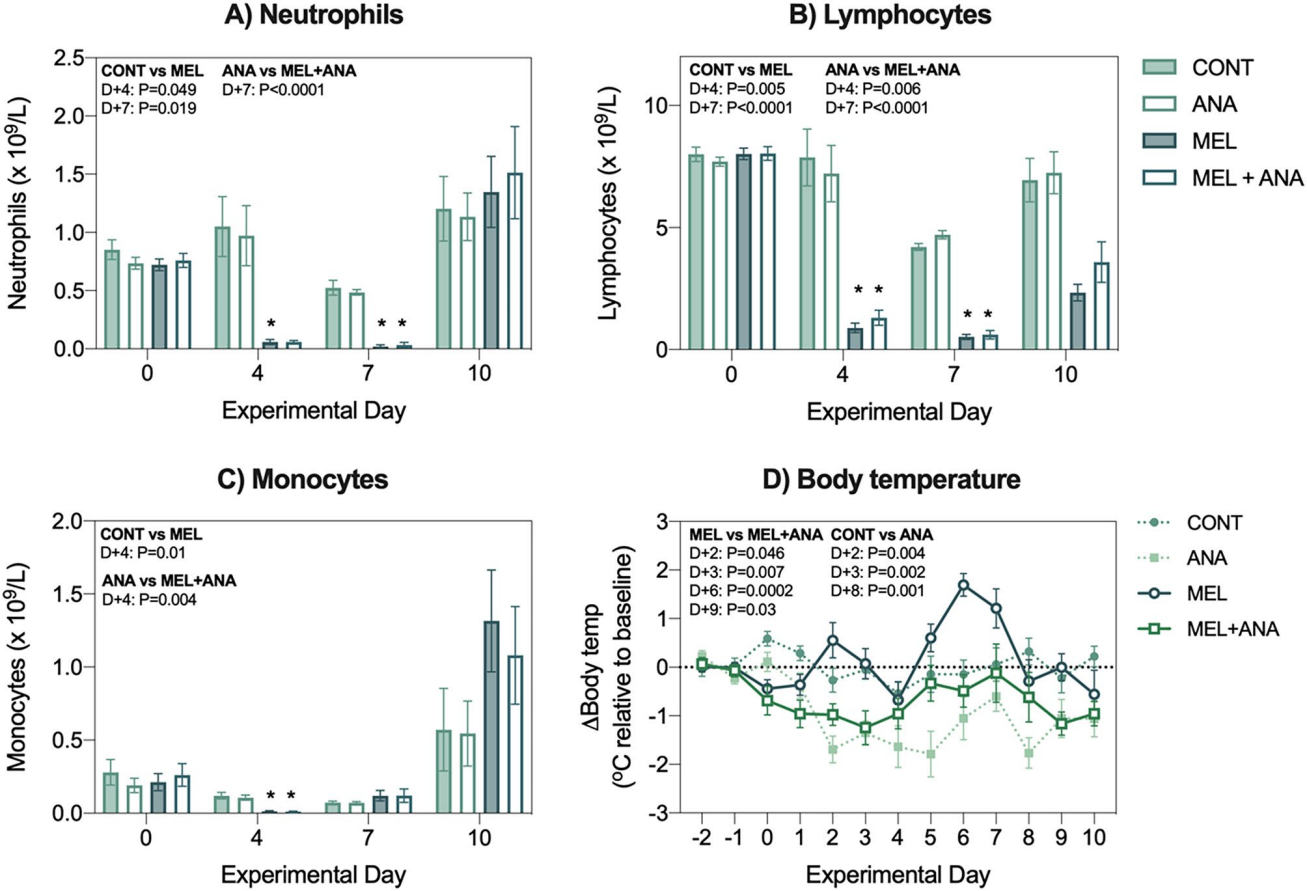
Anakinra does not impair the myeloablative properties of melphalan but controls fever. (**A**) Neutrophils, (**B**) lymphocytes and (**C**) monocytes assessed in whole blood at termination. Melphalan induced fever (**D**), which was controlled by anakinra. Data presented as mean ± SEM. N = 8/group with all data analyzed using mixed-effects model with Geisser-Greenhouse correction for multiple comparisons.

Melphalan caused a significant decrease in total white blood cells, driven by depletion of neutrophils, lymphocytes and monocytes *P* < 0.02; Fig. 3A–C). Anakinra did not influence the degree or duration of neutropenia, lymphopenia or monocytopenia caused by melphalan.

Melphalan caused a significant increase in body temperature (*P* = 0.004, Fig. 3D) indicative of fever. Anakinra, when administered alone and in combination with melphalan, caused a significant decrease in body temperature relative to controls and MEL only animals (*P* < 0.003, *P* < 0.05, respectively). Anakinra prevented fever in the acute phases of MBI (*P* < 0.04).

#### Anakinra controls enteric pathogen expansion following melphalan

**Figure 4:**
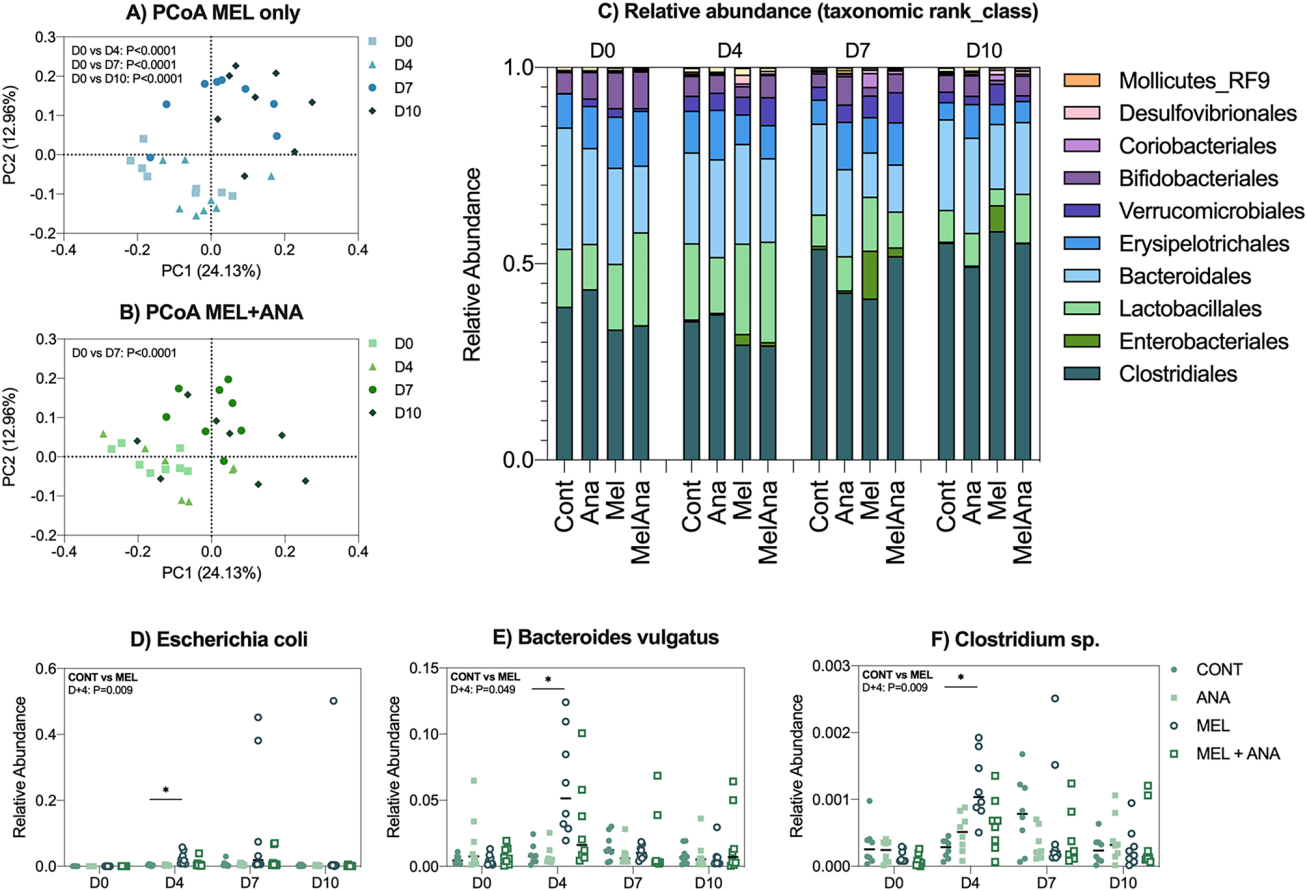
Anakinra reduces mucosal barrier injury-associated dysbiosis and controls enteric pathogen expansion. Melphalan caused compositional changes in the fecal microbiota that persisted for the entire experimental period (**A**). MEL + ANA rats only showed significant PCoA changes at day 4 (**B**). Taxonomic analysis (**C**) showed expansion of Enterobacteriales in MEL only rats, with increases in E.Coli (**D**), Bacteroidetes vulgates (**E**) and Clostridium sp (**F**). Data shown as individual samples, with the exclusion of Panel C which shows mean relative abundance. N = 8/group, all analyzed longitudinally via repeated stool collection. All statistical analyses for microbiota data are outlined in supplementary methods.

Our model of melphalan-induced MBI causes significant microbial disturbances characterised by the expansion of enteric pathogens largely belonging to the *Proteobacteria* phylum. This resulted in a detectable compositional shift that remained significant for the entire study duration (PCoA Fig. 4A). In contrast, MEL + ANA were protected from acute (day 4) and chronic (day 10) microbial injury, with only a significant difference identified at day 7 (Fig. 4B, *P* < 0.0001). Compositionally, MEL + ANA showed mild expansion of *Enterobacteriales* (Fig. 4C) but were largely protected from melphalan-induced pathogen expansion (Fig. 4D–F).

**Figure 5:**
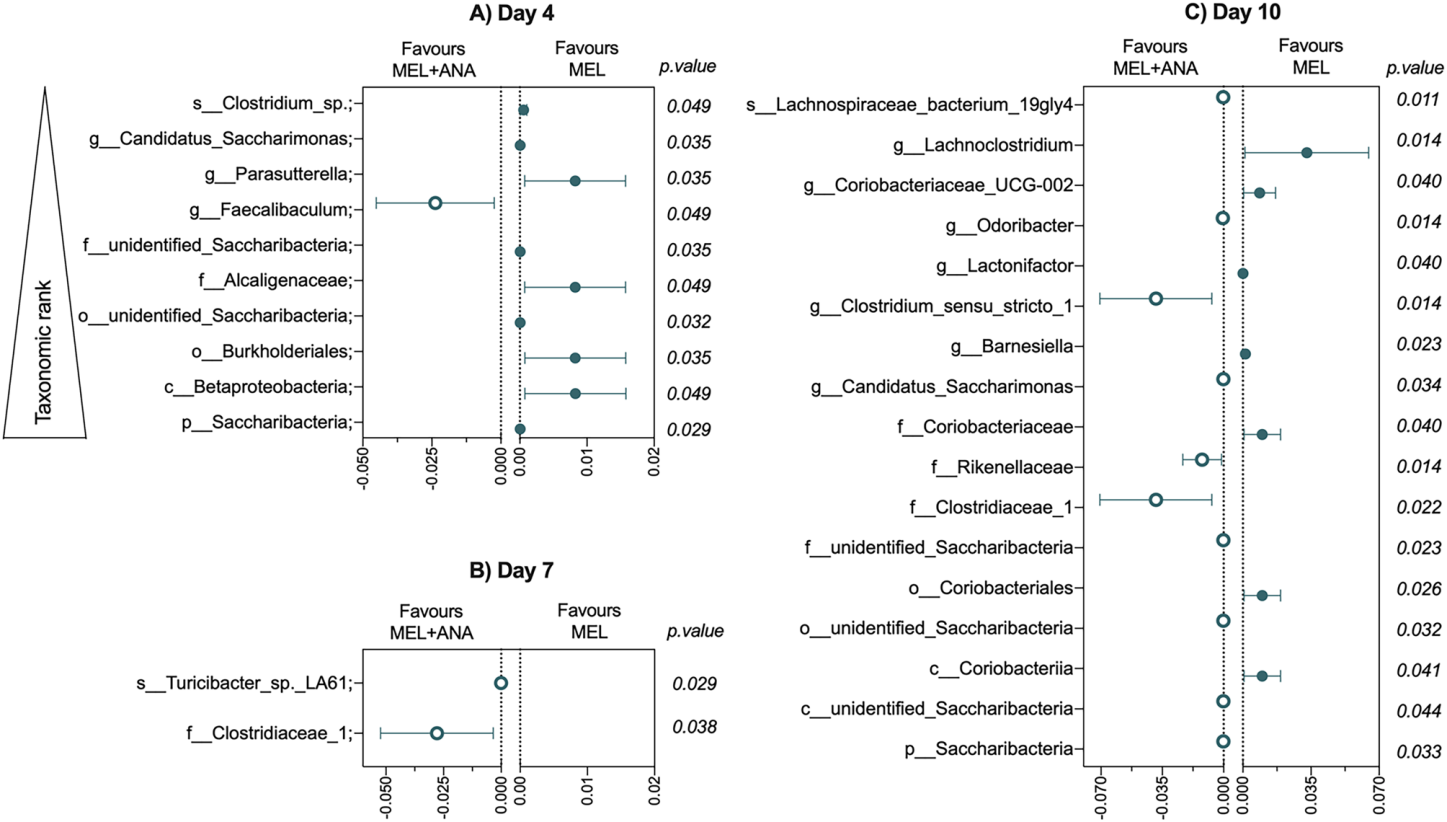
Anakinra promotes commensal stability and controls pathogen expansion. Melphalan alone depleted key commensal microbes and caused expansion of pathogens at day 4 (**A**), 7 (**B**) and 10 (**C**), each of which were minimized by anakinra. Data show median difference between groups (median ± 95% CI). All statistical analyses for microbiota data are outlined in supplementary methods.

When comparing relative abundance data for MEL vs MEL + ANA, anakinra promoted a more stable microbiota with increased relative abundance of *Faecalibaculum* at D + 4 (*P* = 0.049) and control of pathogen expansion (Fig. 5, Table 1). Of note, anakinra induced several differences in the baseline microbial phenotype, with mild but significant increases in the *Firmicutes* and *Bacteroidetes* phyla (Fig. S2).

**Table 1:** Relative abundance of significantly altered microbial taxa in MEL and MEL + ANA treatment groups.

	Taxa	MEL		MEL + ANA		
		Mean	SD	Mean	SD	P
Day 4	k__Bacteria;p__Saccharibacteria;	4.69E-05	3.31E-05	1.63E-05	2.14E-05	0.049
	k__Bacteria;p__Proteobacteria;c__Betaproteobacteria;	0.011	0.008	0.003	0.001	0.035
	k__Bacteria;p__Proteobacteria;c__Betaproteobacteria;o__Burkholderiales;	0.011	0.008	0.003	0.001	0.035
	k__Bacteria;p__Saccharibacteria;c__unidentified_Saccharibacteria;o__unidentified_Saccharibacteria;	4.69E-05	3.31E-05	1.63E-05	2.14E-05	0.049
	k__Bacteria;p__Proteobacteria;c__Betaproteobacteria;o__Burkholderiales;f__Alcaligenaceae;	0.011	0.008	0.003	0.001	0.035
	k__Bacteria;p__Saccharibacteria;c__unidentified_Saccharibacteria;o__unidentified_Saccharibacteria;f__unidentified_Saccharibacteria;	4.69E-05	3.31E-05	1.63E-05	2.14E-05	0.049
	k__Bacteria;p__Firmicutes;c__Erysipelotrichia;o__Erysipelotrichales;f__Erysipelotrichaceae;g__Faecalibaculum;	0.022	0.014	0.046	0.023	0.032
	k__Bacteria;p__Proteobacteria;c__Betaproteobacteria;o__Burkholderiales;f__Alcaligenaceae;g__Parasutterella;	0.011	0.009	0.003	0.001	0.035
	k__Bacteria;p__Saccharibacteria;c__unidentified_Saccharibacteria;o__unidentified_Saccharibacteria;f__unidentified_Saccharibacteria;g__Candidatus_Saccharimonas;	4.69E-05	3.31E-05	1.63E-05	2.14E-05	0.049
	k__Bacteria;p__Firmicutes;c__Clostridia;o__Clostridiales;f__Lachnospiraceae;g__unidentified_Lachnospiraceae;s__Clostridium_sp.;	0.001	0.005	0.0006	0.0004	0.029
Day 7	k__Bacteria;p__Firmicutes;c__Clostridia;o__Clostridiales;f__Clostridiaceae_1;	0.018	0.011	0.046	0.028	0.029
	k__Bacteria;p__Firmicutes;c__Erysipelotrichia;o__Erysipelotrichales;f__Erysipelotrichaceae;g__Turicibacter;s__Turicibacter_sp._LA61;	1.22E-05	1.90E-05	3.47E-05	2.03E-05	0.038
Day 10	k__Bacteria;p__Saccharibacteria;	1.43E-05	1.36E-05	0.00012231	9.02E-05	0.011
	k__Bacteria;p__Saccharibacteria;c__unidentified_Saccharibacteria;	1.43E-05	1.36E-05	0.00012027	9.30E-05	0.014
	k__Bacteria;p__Actinobacteria;c__Coriobacteriia;	0.014	0.011	0.004	0.003	0.040
	k__Bacteria;p__Saccharibacteria;c__unidentified_Saccharibacteria;o__unidentified_Saccharibacteria;	1.43E-05	1.36E-05	0.00012027	9.30E-05	0.014
	k__Bacteria;p__Actinobacteria;c__Coriobacteriia;o__Coriobacteriales;	0.015	0.011	0.004	0.003	0.040
	k__Bacteria;p__Saccharibacteria;c__unidentified_Saccharibacteria;o__unidentified_Saccharibacteria;f__unidentified_Saccharibacteria;	1.43E-05	1.36E-05	0.00012027	9.30E-05	0.014
	k__Bacteria;p__Firmicutes;c__Clostridia;o__Clostridiales;f__Clostridiaceae_1;	0.025	0.014	0.063	0.037	0.023
	k__Bacteria;p__Bacteroidetes;c__Bacteroidia;o__Bacteroidales;f__Rikenellaceae;	0.006	0.002	0.018	0.013	0.034
	k__Bacteria;p__Actinobacteria;c__Coriobacteriia;o__Coriobacteriales;f__Coriobacteriaceae;	0.015	0.011	0.005	0.003	0.040
	k__Bacteria;p__Saccharibacteria;c__unidentified_Saccharibacteria;o__unidentified_Saccharibacteria;f__unidentified_Saccharibacteria;g__Candidatus_Saccharimonas;	1.43E-05	1.36E-05	0.00012027	9.30E-05	0.014
	k__Bacteria;p__Bacteroidetes;c__Bacteroidia;o__Bacteroidales;f__Porphyromonadaceae;g__Barnesiella;	0.002	0.001	0.0005	0.0003	0.022
	k__Bacteria;p__Firmicutes;c__Clostridia;o__Clostridiales;f__Clostridiaceae_1;g__Clostridium_sensu_stricto_1;	0.025	0.014	0.064	0.037	0.023
	k__Bacteria;p__Firmicutes;c__Clostridia;o__Clostridiales;f__Lachnospiraceae;g__Lactonifactor;	4.08E-05	3.90E-05	2.04E-06	5.77E-06	0.026
	k__Bacteria;p__Bacteroidetes;c__Bacteroidia;o__Bacteroidales;f__Porphyromonadaceae;g__Odoribacter;	0.0002	0.0001	0.0007	0.0006	0.032
	k__Bacteria;p__Actinobacteria;c__Coriobacteriia;o__Coriobacteriales;f__Coriobacteriaceae;g__Coriobacteriaceae_UCG-002;	0.011	0.009	0.002	0.004	0.041
	k__Bacteria;p__Firmicutes;c__Clostridia;o__Clostridiales;f__Lachnospiraceae;g__Lachnoclostridium;	0.046	0.035	0.013	0.020	0.044
	k__Bacteria;p__Firmicutes;c__Clostridia;o__Clostridiales;f__Ruminococcaceae;g__Ruminococcaceae_NK4A214_group;s__Lachnospiraceae_bacterium_19gly4;	0.0002	0.0001	0.0005	0.0003	0.033

### Phase IIA study

#### Anakinra showed no safety issues in HSCT recipients treated with HDM

##### Patients and anakinra dosing

We recruited nine participants (N = 9) as planned into our Phase IIA clinical study running between April 2018 and April 2019 at the Radboud University Medical Center. Patient demographics are outlined in Table 2. All patients were treated consecutively with anakinra, with each cohort consisting of three patients (100, 200 and 300 mg). Cohort expansion was not indicated, as no DLTs were reported. The maximum tolerated dose of anakinra was 300 mg.

**Table 2:** Patient demographics and bacteremia data AFFECT-1 Phase IIA trial.

Group	100 mg Anakinra			200 mg Anakinra			300 mg Anakinra		
Patient	1	2	3	4	5	6	7	8	9
Sex	F	M	M	M	M	F	M	F	M
Age (years)	50	68	60	67	66	66	55	64	57
BMI	33.43	27.10	27.49	27.59	26.52	24.34	23.66	28.15	24.11
ECOG	Grade 1	Grade 1	Grade 0	Grade 1	Grade 1	Grade 1	Grade 0	Grade 2	Grade 0
MM stage (ISS)	Stage I	Stage II	Stage I	Stage II	Stage II	Stage I	Stage II	Stage III	Stage II
Hospital stay (days)	20	16	27	19	20	20	21	25	19
Days to engraftment	14	18	19	15	16	13	20	14	14
First occurrence of fever	Day + 6	Day + 9	Day + 13	NA	Day + 11	Day + 8	Day + 11	Day + 6	Day + 9
Bacteremia	Staphylococcus hominis (Day + 7, + 8, + 10)	Negative	Negative	Negative	Negative	Haemolytic streptococcus group C (Day + 8/ + 9)	Negative	Negative	Streptococcus mitis (Day + 9)
CRBSI	Yes (Staphylococcus hominis)	No	No	No	No	No	No	No	No
Use of antibiotics	Ceftazidime (Day + 5 to + 11) Teicoplanin (Day + 11 to + 16)	Ceftazidime (Day + 7 to + 12)	Ceftazidime (Day + 13 to + 18) Piperacillin/ Tazobactam (Day + 20 to + 23)	no	Ceftazidime Day + 11 to + 15)	Ceftazidime (Day + 8 to + 14)	Ceftazidime (Day + 11 to + 16)	Ceftazidime (Day + 6 to + 13)	Ceftazidime (Day + 9 to + 14)

##### Safety and primary endpoint

No SAEs or DLTs occurred in this study. Anakinra was tolerated well in all patients. The most common adverse events grade 3–4 included nausea, diarrhea, and fever during neutropenia and did not differ to toxicities typically seen in patients treated with HDM. One patient developed a central-line related thrombosis, which was deemed to be unrelated to treatment with anakinra.

##### Engraftment, fever and laboratory analyses

**Figure 6:**
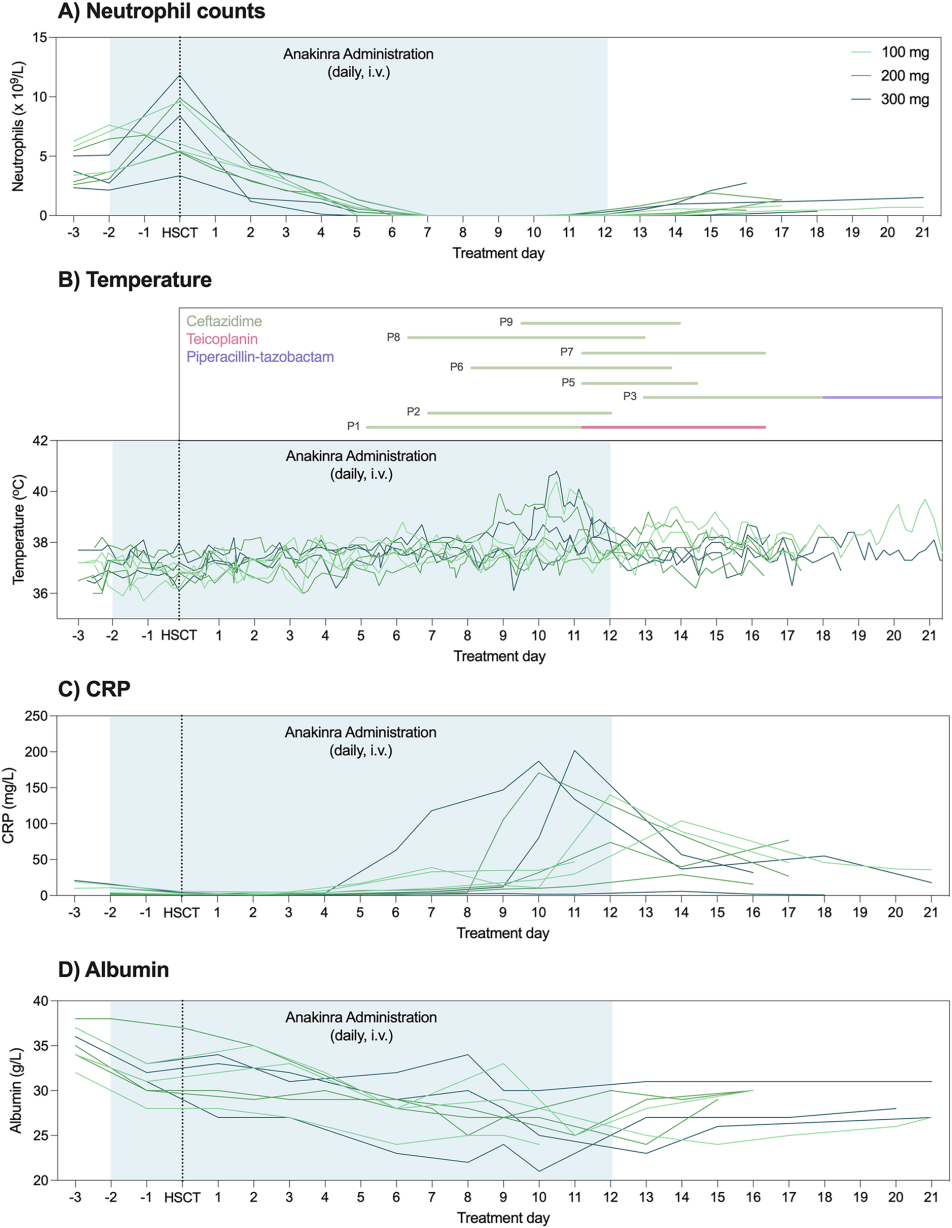
Clinical assessments in Phase IIA AFFECT-1 cohort treated with 100, 200 or 300 mg anakinra. (**A**) Neutrophils, (**B**) temperature with data on antbiotic use, (**C**) CRP and (**D**) albumin were assessed longitudinally in all participants. No differences were detected in any parameter. Data shown as individual participant trajectories.

Time to neutrophil recovery was not prolonged in our patient cohort (15.3^13–19^ days, Fig. 6A). Of nine treated patients, eight developed fever, one of which had fever on only one time point. The day of occurrence of fever was between day + 6 (T = 10) and day + 13 (T = 17) (Fig. 6B), with average temperatures for each group comparable (100 mg: 37.68 ± 0.71 °C, 200 mg: 37.58 ± 0.63 °C, 300 mg: 37.67 ± 0.59 °C). Four patients developed bacteremia. The cultured microorganisms were *Staphylococcus hominis*, *Haemolytic Streptococcus group C* and *Streptococcus mitis.* Catheter-related BSI (CRBSI) was seen in one patient, in which *Staphylococcus hominis* was cultured.

Laboratory analysis of hematological and chemistry panels showed abnormalities typical of HDM conditioning in HSCT (Fig. 6C/D). These included electrolyte disturbances such as hypoalbuminemia, hypokalemia and hypophosphatemia.

##### Exploratory analyses

Systemic cytokine analysis showed a dose-dependent response in IL-1RA, CXCL1 and IL-10 (Fig. S4). Microbiome analyses showed no clear dose–response for OTU and alpha diversity (Fig. S5).

## Discussion

Mucosal barrier injury (MBI) in the gastrointestinal tract remains a major clinical obstacle in the effective treatment of hematological malignancies, driving local and systemic complications that negatively impact treatment outcomes. Here, we provide the first evidence of hyper-activation of the IL-1β/CXCL1/neutrophil axis as a major driver of MBI (induced by melphalan), which supports evaluating the IL-1RA anakinra, both preclinically and clinically. Our data reinforce that strengthening the mucosal barrier with anakinra is safe and effective in controlling MBI which in turn, stabilises the host microbiota and minimises febrile events. Together, these findings represent a significant advance in prompting new therapeutic initiatives that prioritise maintenance of the gut microenvironment.

The IL-1β/CXCL1/neutrophil axis is documented to drive intestinal mucosal inflammation, activated by ligation of intestinal pattern recognition receptors, including toll-like receptors (TLRs)^31^. In the context of MBI, TLR4 activation is known to drive intestinal toxicity^32, 33^, however targeting TLR4 directly is challenging due to emerging regulation of tumour response^34–37^. As such, we selected anakinra as our intervention to inhibit inflammatory mechanisms downstream of TLR4. While anakinra was able to minimise the intensity and duration of MBI, it did not completely prevent it with comparable citrulline dynamics across animal groups in the first 48 h after melphalan treatment. This reflects the core pathobiological understanding of MBI which is initiated by direct cytotoxic events which activate a cascade of inflammatory signalling that serve to exacerbate mucosal injury and the subsequent breakdown of the mucosal barrier^33^. By preventing this self-perpetuating circle of injury with anakinra, we were able to effectively minimise the duration of MBI and thus have a profound impact on the clinical symptomology associated with MBI including weight loss and anorexia. These findings firstly highlight the cluster of (pre-)clinical symptoms related to MBI (malnutrition, anorexia, diarrhea)^38^ and suggest that the mucoprotective properties of anakinra will provide broader benefits to the host, mitigating the need for intensive supportive care interventions (e.g. parenteral nutrition).

In line with our hypothesised approach, minimising the duration of MBI reduced secondary events including enteric pathobiont expansion and fever. This again reiterates that changes in the host microbiome and associated complications can be controlled by strengthening the mucosal barrier^39^. It can be postulated that by minimising the intensity of mucosal injury, the hostility of the microbial environment is reduced ensuring populations of commensal microbes to be maintained. This is supported by our results with the abundance of *Faecalibaculum* maintained throughout the time course of MBI. *Faecalibaculum* is a potent butyrate-producing bacterial genus documented to control pathogen expansion by acidification of the luminal environment. Administration of *Faecalibacteria prausnitzii* has been shown to reduce infection load in a model of antibiotic-induced *Clostridioides difficile* infection, whilst also showing mucoprotective benefits in models of MBI^40, 41^. Furthermore, it is documented to cross feed other commensal microbes increasing colonization resistance. Together, these underscore the luminal benefits of strengthening the mucosal barrier and suggest that maintenance of commensal microbes is central to minimizing translocation events and subsequent BSI.

In our clinical Phase IIA study with 3 + 3 design, we have shown that treatment with anakinra, up until a dose of 300 mg, appears to be safe, feasible, and tolerated well. Of course, the sample size of this study was relatively small. However, anakinra was previously evaluated for its efficacy in the treatment of acute and chronic GvHD in patients allogeneic HSCT. In these studies, patients were treated for a similar time period (with higher doses of anakinra). No differences were seen between the anakinra and placebo group regarding (S)AEs, including infections and time to neutrophil recovery. There were no significant changes in our exploratory analyses, however, it was of note to see marked increase in IL-10 in patients that received 300 mg anakinra. This may reflect anakinra’s capacity to promote anti-inflammatory signaling as observed in COVID-19 related respiratory events^42^. However, with our sample size it is not possible to make any conclusions on this mechanism. Our conclusion is that the recommended dose (RP2D) for anakinra is 300 mg QD, which will be investigated in Phase IIB trial (AFFECT-2 study: Anakinra: Efficacy in the Management of Fever During Neutropenia and Mucositis in ASCT; clinicaltrials.gov identifier NCT04099901)^43^.

While encouraging, our data must be viewed in light of some limitations. Most importantly, our animal model purposely did not include any antimicrobials as we aimed to dissect the true contribution of MBI in pathogen expansion and subsequent febrility. While it is unclear if melphalan has a direct cytotoxic effect on the microbiota, it is likely that MBI drives dysbiosis with antibiotics serving to exacerbate these changes, with previous data demonstrating no direct impact of specific chemotherapeutic agents on microbial viability^44^. As such, assuming dysbiosis is secondary to mucosal injury as recently demonstrated^45^, we anticipate that anakinra will still have an appreciable impact on the severity of dysbiosis and may even prompt more protocolised/limited antibiotic use. Similarly, while we used body temperature as an indicator of BSI, we did not culture peripheral blood or mesenteric lymph nodes as was performed in our animal model development. The ability of anakinra to prevent BSI and thus minimise antibiotic use will be best evaluated in AFFECT-2 where routine blood culture is performed. It is also important to consider that we detected episodes of bacteremia in our participants that were likely caused by skin colonizing organisms; a mechanism anakinra will not influence. While these are expected in HSCT recipients, the majority of infectious cases originate from the gut, and we therefore anticipate anakinra’s capacity to strengthen the mucosal barrier will be clinically impactful in our next study. It must also be acknowledged that limited mechanistic investigations were conducted to identify the way in which anakinra provided mucoprotection. It is well documented that MBI is highly multifactorial, involving mucosal, microbial and metabolic dysfunction^33, 46^; each of which is mediated through aberrant cytokine production. It is therefore unlikely that anakinra will affect distinct pathways, instead dampening multiple mechanisms. In translating this evidence to the clinic, the impact of anakinra on symptom control is of greater significance than mechanistic insight.

In conclusion, we have demonstrated that not only is anakinra safe in HSCT recipients treated with HDM, but may also be an effective strategy to prevent acute MBI. Our data are critical in supporting new antibiotic stewardship efforts directed at mitigating the emerging consequences of antibiotic use. We suggest that minimizing the severity and duration of MBI is an important aspect of infection control that may optimize the efficacy of anti-cancer treatment, decreasing its impact on antibiotic resistance and the long-term complications associated with microbial disruption.

### Methods

#### Preclinical model

This study is reported using the ARRIVE guidelines for the accurate and reproducible reporting of animal research.

##### Ethical statement and animal husbandry

All animal studies were approved by the Dutch Centrale Commissie Dierproeven (CCD) and the Institutional Animal Care and Use Committee of the University Medical Centre Groningen, University of Groningen (RUG), under the license number 171325-01(-002). The procedures were carried out in accordance with the Dutch Experiments on Animals (Wet op de Dierproeven) and the EU Directive 2010/63/EU. All animals were individually housed in conventional, open cages at the Centrale Dienst Proefdieren (CDP; Central Animal Facility) at the University Medical Centre Groningen. Rats (single housed) were housed under 12 h light/dark cycles with ad libitum access to autoclaved AIN93G rodent chow and sterile water. All rats acclimatised for 10 days and randomised to their treatment groups via a random number sequence generated in Excel. Small adjustments were made to ensure comparable body weight at the time of treatment and cages were equally distributed across racks to minimise confounding factors. HRW was responsible for animal allocation and assessments while RH/ARDSF performed treatments. Softened chow and subcutaneous saline were provided to rats to reduce suffering/distress and were humanely euthanised if a clinical toxicity score > / = 12 was observed. This score was calculated based on weight loss, diarrhea, reluctance to move, coat condition and food intake; each of which were assessed 0–3. At completion of the study, rats were anaesthetised with 5% isoflurane in an induction chamber, followed by cardiac puncture and cervical dislocation (isoflurane provided by a facemask).

##### Model development and cytokine assessment

We have previously reported on the development and validation of our HDM model of MBI, which exhibits both clinical and molecular consistency with patients undergoing HDM treatment^21^. During model development, plasma (isolated from whole blood) was collected and stored for cytokine analysis to inform the selection of our intervention. Repeated whole blood samples (75 µl) were collected from the tail vein into EDTA-treated haematocrit capillary tubes on day 0, 4, 7 and 10.

Cytokines (IFN-γ, IL-1β, IL-4, IL-5, IL-6, IL-10, IL-13, KC/GRO and TNF-α) using the Meso Scale Discovery V-Plex Proinflammatory Panel Rat 2 following manufacturer’s guidelines. On the day of analysis, all reagents were brought to room temperature, samples were centrifuged to remove any particulate matter and diluted 1:4. Data analysis was performed using the Meso Scale Discovery Workbench.

##### Intervention study design

Male albino Wistar rats (150–180 g) were randomized (Excel number generator) to one of four experimental groups (N = 16/group): (1) controls (phosphate buffered saline (PBS) + 0.9% NaCl), (2) anakinra + 0.9% NaCl, (3) PBS + melphalan, and (4) anakinra + melphalan. Melphalan was administered as a single, intravenous dose on day 0 (5 mg/kg, 10 mg/ml) via the penile vein under 3% isoflurane anaesthetic. Anakinra was administered subcutaneously (100 mg/kg, 150 mg/ml) twice daily from day − 1 to + 4 (8 am and 5 pm). N = 4 rats per group were terminated at the exploratory time points (day 4, and 7) and N = 8 on day 10 (recovery phase) by isoflurane inhalation (3%) and cervical dislocation. The primary endpoint for the intervention study was plasma citrulline, a validated biomarker of MBI^19, 47^, which was used for all power calculations (N = 8 required, alpha = 0.05, beta = 0.8).

##### Clinical toxicity assessment

Clinical manifestations of MBI were assessed using validated parameters of body weight, food intake and water intake, as well as routine welfare indicators (movement, posture, coat condition). Rats were weighed daily, and water/food intake monitored by manual weighing of chow and water bottles.

##### Plasma citrulline

Plasma citrulline is an indicator of intestinal enterocyte mass^48^, and a validated biomarker of intestinal MBI. Repeated blood samples (75 µl) were collected from the tail vein into EDTA-treated haematocrit capillary tubes on day 0, 2, 4, 6, 7, 8 and 10. Citrulline was determined in 30 μl of plasma (isolated from whole blood via centrifugation at 4000 g for 10 min) using automated ion exchange column chromatography as previously described^49^.

##### Differential blood analysis

Whole blood samples (200 μl) were collected from the tail vein into MiniCollect® EDTA tubes on day 0, 4, 7 and 10 for differential morphological analysis which included: white blood cell count (WBC, 10^9^/L), red blood cell count (RBC, 10^9^/L), haemoglobin (HGB, mmol/L), haematocrit (HCT, L/L), mean corpuscular volume (MCV, fL), mean corpuscular haemoglobin (MCH, amol), mean corpuscular hemoglobin concentration (MCHC, mmol/L), platelet count (PLT, 10^9^/L), red blood cell distribution width (RDW-SD/-CV, fL/%), mean platelet volume (fL), mean platelet volume (MPV, fL), platelet large cell ratio (P-LCR, %), procalcitonin (PCT, %), nucleated red blood cell (NRBC, 10^9^/L and %), neutrophils (10^9^/L and %), lymphocytes (10^9^/L and %), monocytes (10^9^/L and %), eosinophils (10^9^/L and %), basophils (10^9^/L and %) and immunoglobulins (IG, 10^9^/L and %). For the purpose of the current study only neutrophils, lymphocytes and monocytes were evaluated.

##### Body temperature

Core body temperature was used as an indicator of fever. Body temperature was assessed daily using the Plexx B.V. DAS-7007R handheld reader and IPT programmable transponders. Transponders were inserted subcutaneously under mild 2% isoflurane anaesthesia on day − 4. Average values from day − 4 to − 1 were considered as baseline body temperature.

##### Microbiota analysis

The microbiota composition was assessed using 16S rRNA sequencing in N = 8 rats/group. Repeated faecal samples were collected on day 0, 4, 7 and 10 and stored at − 80 °C until analysis. Sample preparation (including DNA extraction, PCR amplification, library preparation), quality control, sequencing and analyses were all performed by Novogene (please see supplementary methods for full description).

##### Statistical analyses

All data (excluding 16S data) were analysed in GraphPad Prism (v8.0. Repeated measures across multiple groups were assessed by mixed-effect models with appropriate post-hoc analyses. Terminal data analyses were assessed by one-way ANOVA. Statistical analyses are outlined in figure legends and *P* < 0.05 was considered significant.

#### Phase IIA trial

##### Goal

This Phase IIA trial (AFFECT-1: NCT03233776, 17/6/2017) aimed to i) assess the safety of anakinra in autologous HSCT recipients undergoing conditioning with HDM, and ii) determine the maximum tolerated dose of anakina (100, 200 or 300 mg).

##### Ethics and patients

This study was approved by the ethical committee Nijmegen-Arnhem (NL59679.091.16; EudraCT 2016-004,419-11) and performed in accordance with (a) the Declaration of Helsinki (1964, amended October 2013), (b) Medical Research Involving Human Subjects Act and c) Good Clinical Practice guidelines. We enrolled patients from Radboud University Medical Centre who were at least 18 years of age and were scheduled to undergo an autologous HSCT after receiving conditioning with HDM (200 mg/m^2^) for multiple myeloma. All participants provided informed consent. Important exclusion criteria were active infections, a history of tuberculosis or positive Quantiferon, glomular filtration rate < 40 ml/min, and colonization with highly resistant micro-organisms or with gram-negative bacteria resistant to ciprofloxacin.

##### Patient involvement

Patients were involved in the design of the AFFECT trials, through involvement of *Hematon*, a patient organization for patients with hemato-oncological diseases in the Netherlands. The project plan, including trial materials, have been presented to patient experts from Hematon. They have given their advice on the project, and provided input on the design of the study as well as on patient information. Patients will also be involved in the dissemination of the results of the AFFECT trials. Information on both the design as well as the outcome of the AFFECT trials is and/or will be available on websites specifically aimed at patients, such as the Dutch website ‘kanker.nl’.

##### Treatment protocol

Conforming with routine clinical practice and care, study participants were admitted at day − 3, treated with melphalan 200 mg/m^2^ at day − 2, and received their autologous HSCT at day 0. They were treated with IL-1RA anakinra (Kineret, SOBI) intravenously once daily from day − 2 up until day + 12.

##### Study design

A traditional 3 + 3 design was used (Fig. S1), in which the first cohort of patients was treated with 100 mg, the next cohort with 200 mg and the third cohort with 300 mg of anakinra. In this study design, the cohort is expanded when dose limiting toxicities (DLTs) occur. The primary study endpoint was safety, using the common toxicity criteria (CTCAE) version 4.0^50^, as well as the maximum tolerated dose of anakinra (MTD; 100, 200 or 300 mg). DLTs were defined as the occurrence of (1) an infection due to an opportunistic pathogen (including Pneumocystis jirovecii pneumonia, mycobacterial infections and invasive mould disease), (2) a suspected unexpected serious adverse reaction (SUSAR), (3) severe non-hematological toxicity grade 3–4 (meaning toxicity that does not commonly occur in the treatment with HDM and HSCT, or that is more severe than is to be expected with standard treatment) and (4) primary graft failure or prolonged neutropenia (neutrophils have not been > 0.5 × 10^9^/l on one single day, assessed on day + 21, and counting from day 0).

Secondary endpoints included: incidence of fever during neutropenia (defined as a tympanic temperature ≥ 38.5 °C and an absolute neutrophil count (ANC) < 0.5 × 109/l, or expected to fall below 0.5 × 10^9^/l in the next 48 h), CRP levels, intestinal mucositis as measured by (the AUC of) citrulline, clinical mucositis as determined by daily mouth and gut scores, incidence and type of BSI, short term overall survival (100 days and 1 year after HSCT), length of hospital stay in days and use of systemic antimicrobial agents, analgesic drugs and total parenteral nutrition (incidence and duration).

##### Supportive care

Patients received standard antimicrobial prophylaxis including ciprofloxacin and valacyclovir, as well as antifungal prophylaxis (fluconazole) on indication; i.e. established mucosal colonization. Upon occurrence of fever during neutropenia, empirical treatment with ceftazidime was started. The use of therapies to prevent or treat mucositis (i.e. oral cryotherapy) was prohibited. Also, treatment with acetaminophen or non-steroidal anti-inflammatory drugs was not allowed during hospital admission. All other supportive care treatments (i.e. morphine, antiemetics, transfusions, TPN) were allowed.

##### Study procedures

Laboratory analysis was performed three times a week, which included hematological and chemistry panels and plasma collection for citrulline analysis. Blood cultures were drawn daily from day + 4 up until day + 12, which was halted upon occurrence of fever. Outside this period, conforming to standard of care, blood cultures were drawn twice weekly and in occurrence of fever. Conforming standard of care, surveillance cultures of mucosal barriers were obtained twice weekly.

##### Exploratory analyses

Plasma was longitudinally collected from participants throughout the study period for the evaluation of cytokines using the Meso Scale Discovery Customised U-Plex 9-analyte panel following manufacturer’s guidelines (IL-1α/β, IL-1RA, CXCL1, TNFα, IL-10, IL-17, IL-6, GM-CSF). 16S sequencing was performed by Novogene (as per preclinical analysis methodology).

## Supplementary Information

Supplementary Information.

## Data Availability

For access to preclinical data, please contact Dr Hannah Wardill. For clinical data requests, please contact Dr Charlotte de Mooij.
