# Supplementary material for: Supporting the gastrointestinal microenvironment during high-dose chemotherapy and stem cell transplantation by inhibiting IL-1 signaling with anakinra
Source: Sci Rep. 2022 May 11;12:6803. doi: 10.1038/s41598-022-10700-3 (PMC9095632; doi:10.1038/s41598-022-10700-3)
Supplement: Supplementary file 1 — Supplementary Information. [file 41598_2022_10700_MOESM1_ESM.docx]

**Supplementary methods**

**16S rRNA Sequencing by Novogene**

**Sequencing preparation**

*Genomic DNA extraction*

Total genome DNA from samples was extracted using CTAB/SDS method. DNA concentration and purity was monitored on 1% agarose gels. According to the concentration, DNA was diluted to 1ng/μL using sterile water.

*Amplicon Generation*

16S rRNA/18SrRNA/ITS genes of distinct regions (16SV4/16SV3/16SV3-V4/16SV4-V5, 18S V4/18S V9, ITS1/ITS2, Arc V4) were amplified used specific primer (e.g. 16S V4: 515F-806R, 18S V4: 528F-706R, 18S V9: 1380F-1510R) with the barcode. All PCR reactions were carried out with Phusion® High-Fidelity PCR Master Mix (New England Biolabs).

*PCR Products quantification and qualification*

Mix same volume of 1X loading buffer (contained SYB green) with PCR products and operate electrophoresis on 2% agarose gel for detection. Samples with bright main strip between 400bp-450bp were chosen for further experiments.

*PCR Products Mixing and Purification*

PCR products was mixed at equal density ratios. The mixed PCR products were purified with Qiagen Gel Extraction Kit (Qiagen, Germany).

The libraries generated with NEBNext® Ultra^TM^ DNA Library Prep Kit for Illumina and quantified via Qubit and Q-PCR, would be analysed by Illumina platform.

**Information analysis**

*Sequencing data processing*

Paired-end reads was assigned to samples based on their unique barcodes and truncated by cutting off the barcode and primer sequences. Paired-end reads were merged using FLASH (V1.2.7, <http://ccb.jhu.edu/software/FLASH/>) ^[21]^, a very fast and accurate analysis tool, which was designed to merge paired-end reads when at least some of the reads overlap the read generated from the opposite end of the same DNA fragment, and the splicing sequences were called raw tags. Quality filtering on the raw tags were performed under specific filtering conditions to obtain the high-quality clean tags^[22]^ according to the Qiime (V1.7.0, <http://qiime.org/scripts/split_libraries_fastq.html>)^[23]^ quality controlled process. The tags were compared with the reference database (Gold database,<http://drive5.com/uchime/uchime_download.html>) using UCHIME algorithm (UCHIME Algorithm,<http://www.drive5.com/usearch/manual/uchime_algo.html>)^[24]^ to detect chimera sequences (<https://drive5.com/usearch/manual/chimeras.html>). And then the chimera sequences were removed ^[25]^. Then the Effective Tags finally obtained.

*OTU cluster and Taxonomic annotation*

Sequences analysis were performed by Uparse software (Uparse v7.0.1001 <http://drive5.com/uparse/>)^[26]^ using all the effective tags. Sequences with ≥97% similarity were assigned to the same OTUs. Representative sequence for each OTU was screened for further annotation. For each representative sequence, Mothur software was performed against the SSUrRNA database of SILVA Database (<http://www.arb-silva.de/>)^[27]^for species annotation at each taxonomic rank (Threshold:0.8~1)^[28]^ (kingdom, phylum, class, order, family, genus, species). To obtain the phylogenetic relationship of all OTUs representative sequences, the MUSCLE ^[29]^ (Version 3.8.31,<http://www.drive5.com/muscle/>)can compare multiple sequences rapidly. OTUs abundance information were normalized using a standard of sequence number corresponding to the sample with the least sequences. Subsequent analysis of alpha diversity and beta diversity were all performed basing on this output normalized data.

*Alpha Diversity*

Alpha diversity is applied in analyzing complexity of biodiversity for a sample through 6 indices, including Observed-species, Chao1, Shannon, Simpson, ACE, Good-coverage. All these indices in our samples were calculated with QIIME (Version 1.7.0) and displayed with R software (Version 2.15.3).

Alpha Diversity Indices：

Community richness indices:

Chao - the Chao1 estimator (<http://scikit-bio.org/docs/latest/generated/skbio.diversity.alpha.chao1.html#skbio.diversity.alpha.chao1>; ACE - the ACE estimator (<http://scikit-bio.org/docs/latest/generated/skbio.diversity.alpha.ace.html#skbio.diversity.alpha.ace>);

Community diversity indices:

Shannon - the Shannon index (<http://scikit-bio.org/docs/latest/generated/skbio.diversity.alpha.shannon.html#skbio.diversity.alpha.shannon>); Simpson - the Simpson index (<http://scikit-bio.org/docs/latest/generated/skbio.diversity.alpha.simpson.html#skbio.diversity.alpha.simpson>);

The index of sequencing depth:

Coverage - the Good’s coverage (<http://scikit-bio.org/docs/latest/generated/skbio.diversity.alpha.goods_coverage.html#skbio.diversity.alpha.goods_coverage>);

The index of phylogenetic diversity:

PD_whole_tree - PD_whole_tree index(<http://scikit-bio.org/docs/latest/generated/skbio.diversity.alpha.faith_pd.html?highlight=pd#skbio.diversity.alpha.faith_pd>)

*Beta Diversity*

Beta diversity analysis was used to evaluate differences of samples in species complexity, Beta diversity on both weighted and unweighted unifrac were calculated by QIIME software (Version 1.7.0). Cluster analysis was preceded by principal component analysis (PCA), which was applied to reduce the dimension of the original variables using the FactoMineR package and ggplot2 package in R software (Version 2.15.3). Principal Coordinate Analysis (PCoA) was performed to get principal coordinates and visualize from complex, multidimensional data. A distance matrix of weighted or unweighted unifrac among samples obtained before was transformed to a new set of orthogonal axes, by which the maximum variation factor is demonstrated by first principal coordinate, and the second maximum one by the second principal coordinate, and so on. PCoA analysis was displayed by WGCNA package, stat packages and ggplot2 package in R software (Version 2.15.3). Unweighted Pair-group Method with Arithmetic Means (UPGMA) Clustering was performed as a type of hierarchical clustering method to interpret the distance matrix using average linkage and was conducted by QIIME software (Version 1.7.0).

LEfSe analysis was conducted by LEfSe software. Metastat was calculated by R software. P-value was calculated by method of permutation test while q-value was calculated by method of Benjamini and Hochberg False Discovery Rate^[30]^. Anosim, MRPP and Adonis were performed by R software (Vegan package: anosim function, mrpp function and adonis function). AMOVA was calculated by mothur using amova function. T_test and drawing were conducted by R software.

**Supplementary figures and data**

Figures: 5

Tables: 1

**
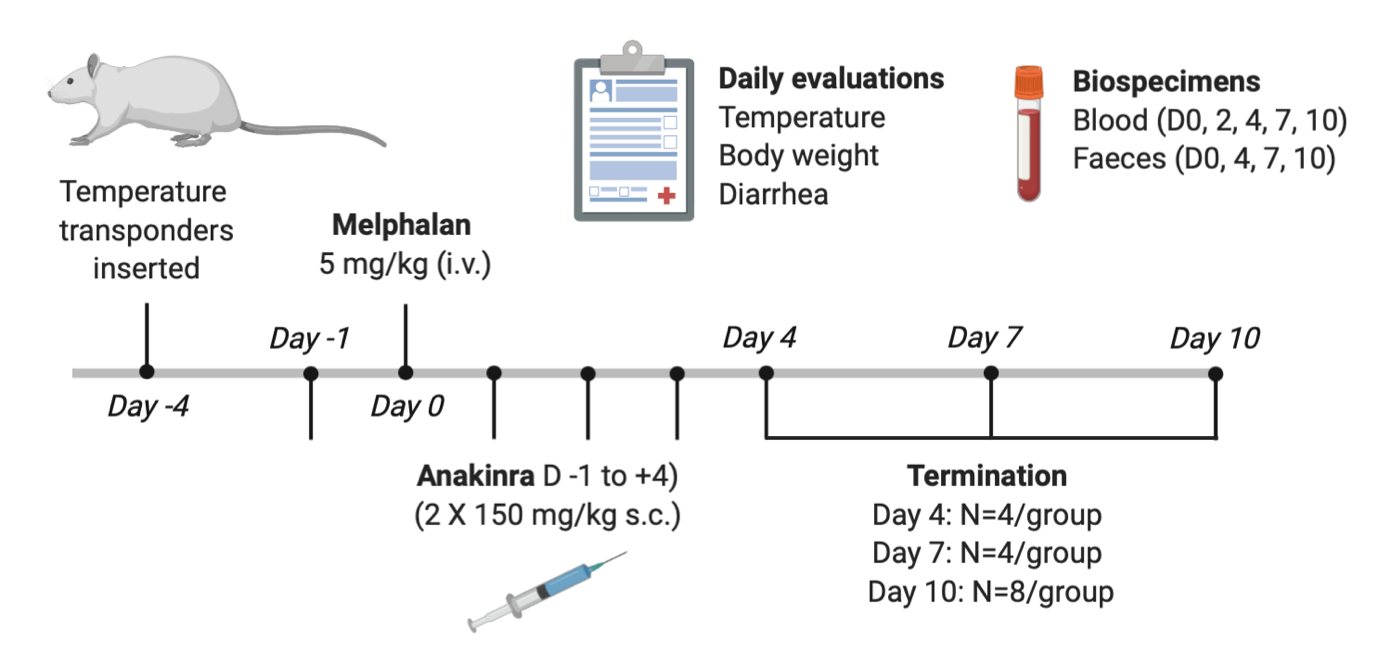
**

**Figure S1 – Experimental design preclinical study. Figure prepared using BioRender.**

**A.**


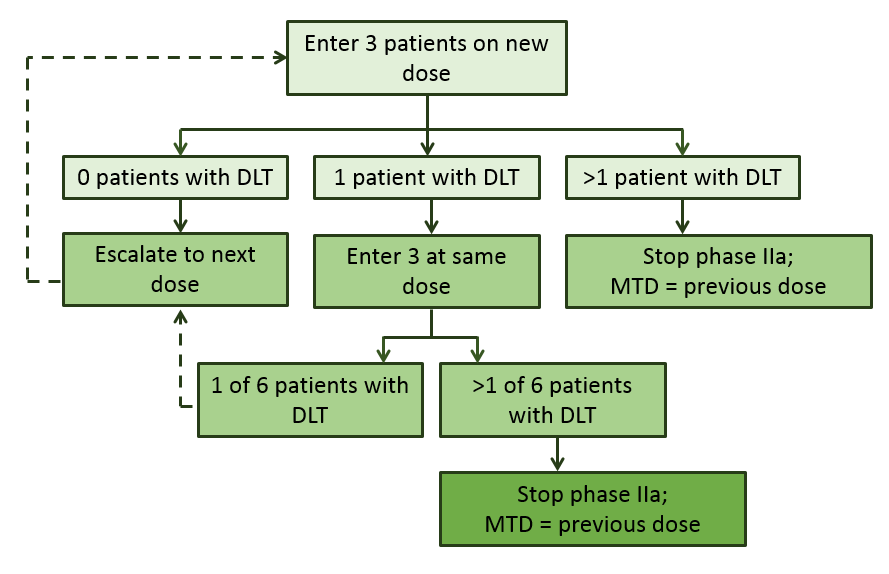


MTD: maximum tolerated dose

DLT: dose-limiting toxicity

**B.**

**
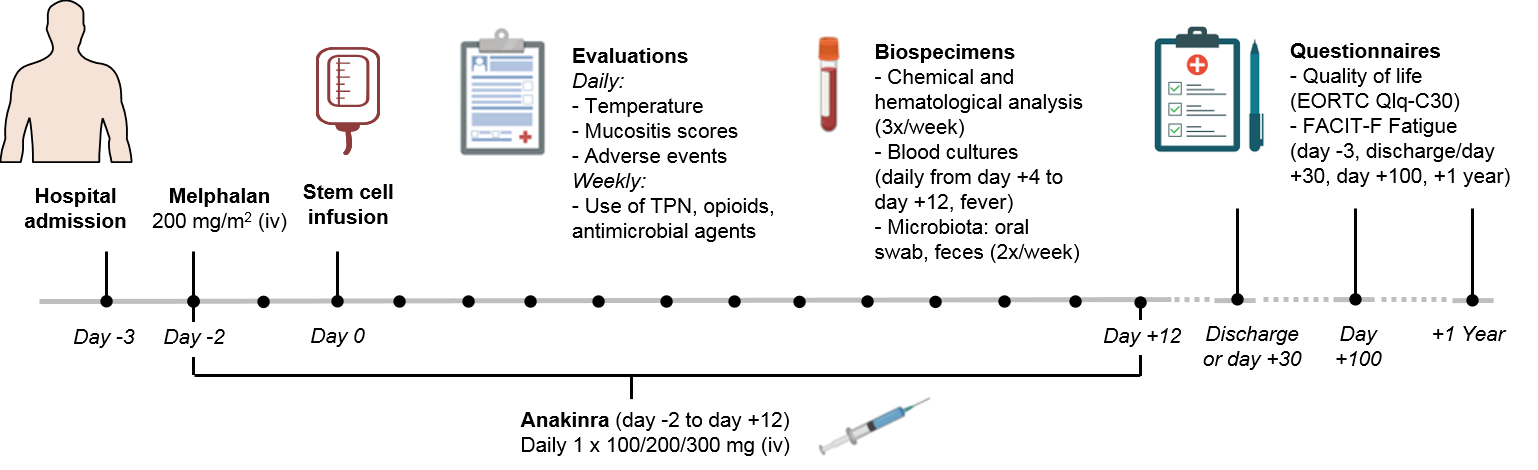
**

**Figure S2 – Experimental design phase IIa clinical study; 3+3 design (A), study design and procedures (B). Figure prepared by C. de Mooij.**


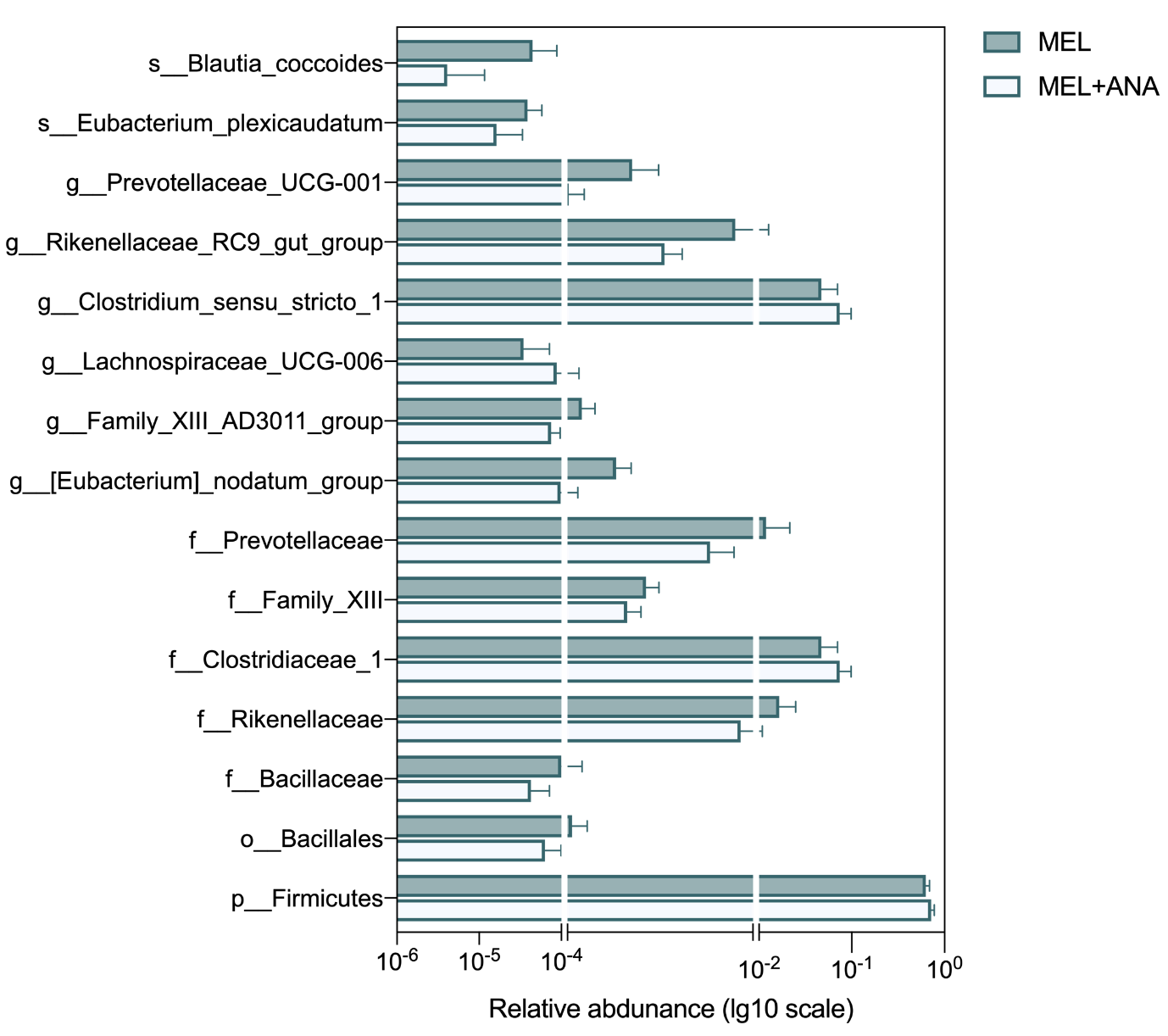


**Figure S3 – Baseline differences in microbiota of MEL vs MEL+ANA rats.**


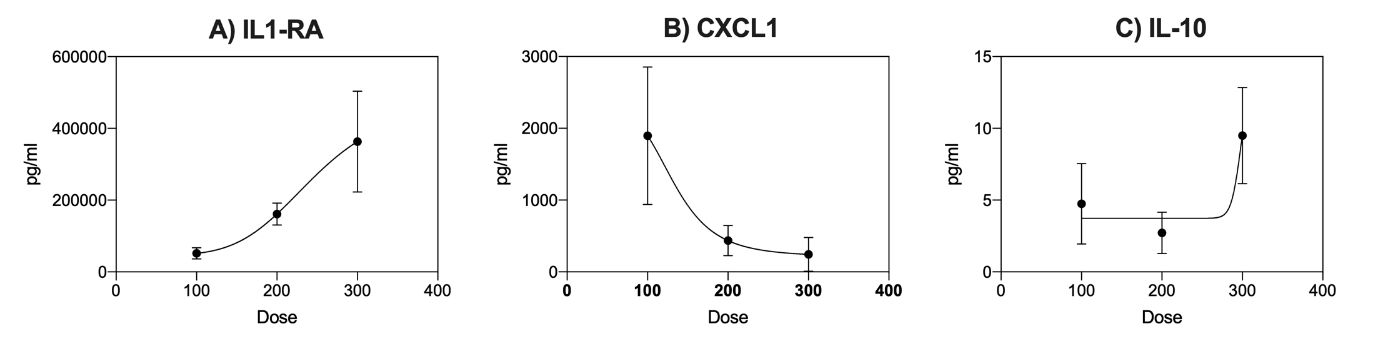


**Figure S4 – Anakinra dose-dependently inhibits CXCL1 and promotes IL-10.**


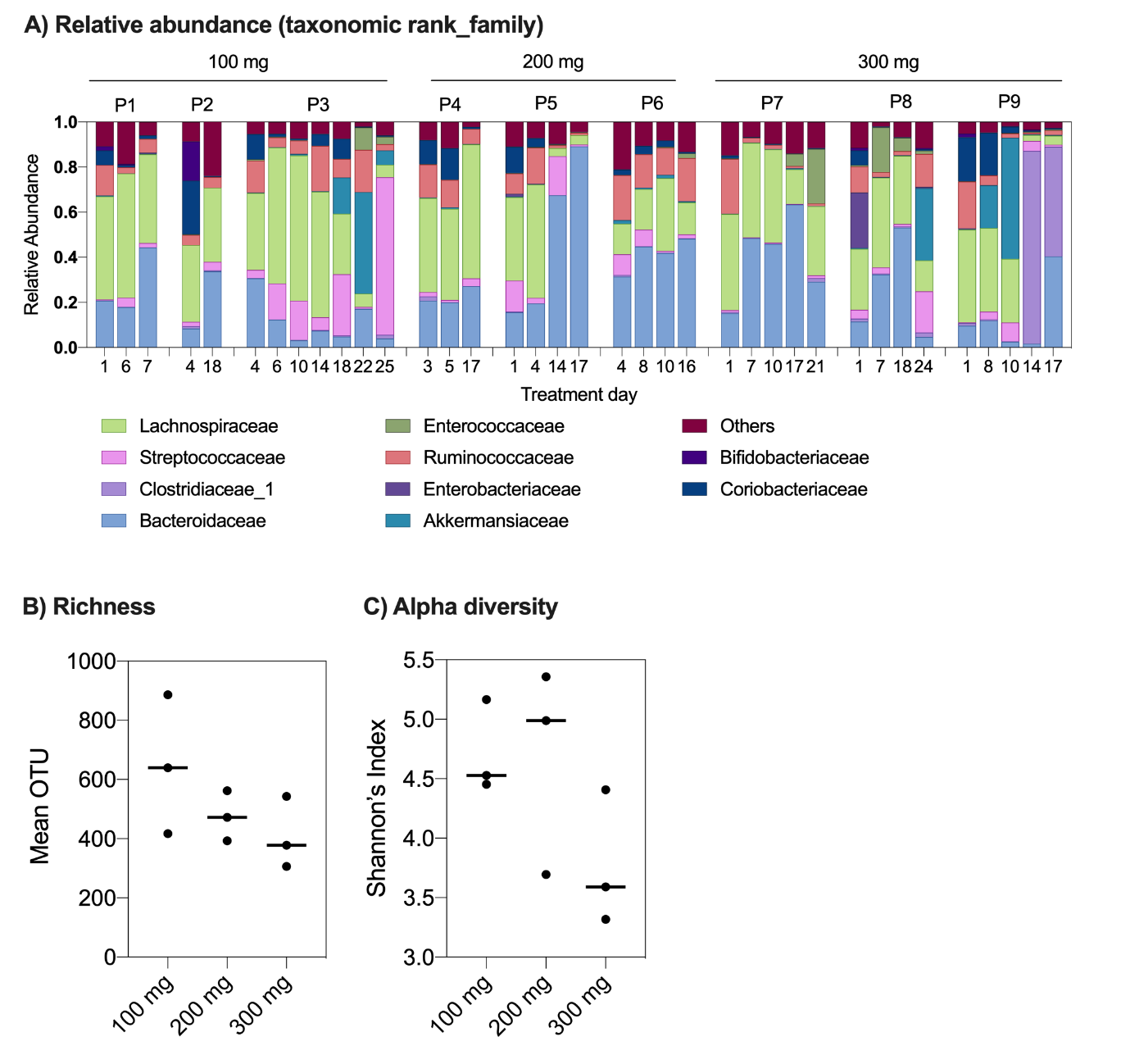


**Figure S5 – Impact of anakinra on the gut microbiota in AFFECT-1 cohort.**

| **Table S1: Baseline data** | | | | | |
| --- | --- | --- | --- | --- | --- |
| Variable | Control | Anakinra | Melphalan | Melphalan + Anakinra | P value |
| Body weight (g) | 191.6 ± 15.1 | 192.6 ± 15.9 | 191.4 ± 9.9 | 192.6 ±16.5 | >0.05 |
| Plasma citrulline (uM) | 90.9 ± 9.5 | 89.9 ± 8.3 | 93.8 ± 11.2 | 91.8 ± 8.3 | >0.05 |
| Daily food intake (g) | 17.3 ± 2.1 | 17.6 ± 1.6 | 17.1 ± 1.0 | 17.1 ± 1.9 | >0.05 |
